# Electrospun Nanofibers Incorporated with HPγCD Inclusion Complex for Improved Water Solubility and Activity of Hydrophobic Fungicides Pyrimethanil

**DOI:** 10.3390/molecules30071456

**Published:** 2025-03-25

**Authors:** Shuang Gao, Honglei Yan, Yue Xiu, Fengrui Li, Yu Zhang, Ruichi Wang, Lixia Zhao, Fei Ye, Ying Fu

**Affiliations:** Department of Chemistry, Northeast Agricultural University, Harbin 150030, China; gaoshuang@neau.edu.cn (S.G.);

**Keywords:** pyrimethanil, hydroxypropyl-γ-cyclodextrin, inclusion complex, electrospinning, nanofibers, hydrophobic pesticide, water solubility

## Abstract

The discovery of efficient and stable nanopesticides with improved water solubility and sustained release effects has become particularly important. Pyrimethanil (Pyr) as a low toxicity fungicide of an aniline pyrimidine group is widely used for the prevention and control of gray mold in crops and ornamental plants, however, poor water solubility hinders its further development. Herein, we use a supramolecular self-assembly process to encapsulate a pyrimethanil in a hydroxypropyl-gamma-cyclodextrin (HPγCD) via electrostatic interactions, thereby constructing the inclusion complex nanofibers. The HPγCD as an environmentally friendly carrier material for pesticide delivery is favorable for facilitating the control efficacy, water solubility, and thermostability with Pyr. The diameter of the prepared inclusion nanofiber is 426.6 ± 82.1 nm. Pyr/HPγCD inclusion complex nanofibers could be completely dissolved in water within 3 s. As predicted, the fungicidal activity of Pyr/HPγCD inclusion complex nanofibers is much higher than that of either Pyr, and the EC_50_ value of Pyr/HPγCD inclusion nanofibers is 0.437 μg/mL, which is about half of that of Pyr (0.840 μg/mL). The inclusion strategy achieved by Pyr and HPγCD is important for improving the safety of nanopesticides. This work provides a versatile insight to promote the development of water-based pesticide dosage forms and reduce pesticide losses in agricultural production.

## 1. Introduction

Pesticides, a vital part of sustainable agricultural development, play a crucial role in plant protection, including the crop protection against weeds, insects, and plant diseases to meet the escalating food demand [1]. Most of the agricultural manufacturers worldwide still depend on the application of pesticides that can reduce losses of 30–40% of total global crop production [2]. However, due to runoff, rebound, and spray drift during pesticide application, less than 0.1 percent of applied pesticides reach their targets [3,4], which impairs the agroecosystem function. Owing to the hydrophobicity of the pesticide’s active components, most pesticides are difficult to directly apply in the field [5]. The hydrophobicity of pesticides has been addressed in traditional pesticides through the addition of organic emulsifiers [6], but organic formulations bring more environmental pollution. With the rapid growth of population, seeking an efficient and green strategy remains important for new pesticide delivery platforms to improve the water solubility and utilization rate of hydrophobic pesticides [7].

The design and fabrication of bicomponent composite-materials might offer a promising delivery platform family towards the pesticide carrier [8]. It is widely employed for preventing the rapid release of pesticides, delaying the degradation of active ingredients, and improving the stability and water solubility of pesticides [3]. This strategy was well established in 2D MXene loaded with abamectin to improve the water solubility and photostability of hydrophobic abamectin [9]. Zhao et al. [10] used glyceryl methacrylate (GMA) linked to carboxymethyl cellulose (CMC) and glycine methyl ester (GLY) to synthesize the pesticide nanocarrier CMC-PGMA-GLY for loading the hydrophobic pesticide methylaminoamyclofenacin benzoate (EB). The prepared EB@CMC-PGMA-GLY had good water dispersibility and leaf wettability. Moreover, the results indicated that the nanocarrier could be utilized as an organic nitrogen fertilizer, which increased the height and fresh weight of crops by 52.56% and 39.77%, respectively. Das’s team [11] firstly constructed an atrazine/hydroxypropyl-β-cyclodextrin inclusion complex (ATZ/HPβCD-IC), then incorporated ATZ/HPβCD-IC into polyvinyl alcohol (PVA) solution, and finally prepared ATZ/HPβCD-IC doped PVA fiber mats (EM) by using the electrospinning technique. The prepared EM improved the thermal stability (from 284 °C to 395 °C) and herbicidal activity of ATZ, and the EM functioned as a slow release for ATZ. Wang et al. [12] used amphiphilic polystyrene-co-maleic anhydride (PSMA) as the pesticide carrier, and prepared nanomicrospheres to load λ-cyfluthrin (PSMA-LC-NS) by using the ultrasonic emulsification-solvent evaporation method. PSMA-LC-NS had relatively high leaf adhesion and excellent hydrophilicity, which provided an idea for the encapsulation of other hydrophobic pesticides. Among the many host molecule materials, natural products have the advantages of simplicity, accessibility, and environmental friendliness [13]. Cyclodextrins (CD), a natural macrocyclic oligosaccharide, show hydrophilic outer surfaces and hydrophobic inner cavities [14]. The special structure of cyclodextrins can encapsulate hydrophobic guest molecules through supramolecular interactions (e.g., hydrophobic interactions, electrostatic interactions, π-π stacking interactions, hydrogen bonding, etc.) to form host–guest inclusion complexes [15]. It is possible to both protect the activity of the guest molecule and improve its properties (e.g., water solubility, thermal stability, photostability, antioxidant activity etc.) [16]. Peng et al. [17] encapsulated Star anise essential oil (SAEO) in β-CD to prepare SAEO and β-CD inclusion compound (SAEO-IC). The MIC value of SAEO-IC to Staphylococcus aureus was 0.78 mg/mL, which was much lower than that of SAEO (1.56 mg/mL) cyclodextrins family, the cavity of γ-cyclodextrin (γ-CD) is the biggest and γ-CD has well-cavity fitting, however, its water solubility is poor [18]. HPγCD as a derivative of γ-CD has a larger cavity, better water solubility, and more easily encapsulates guest molecules with high molecular weight [19].

Electrospinning nanofibers have high porosity, large specific surface area, and play a considerable part in many fields [20], such as agriculture [21], food [22], medicine [23], etc. Based on previous studies, cyclodextrin inclusion complexes can be processed into nanofibers with high porosity and large specific surface area by using electrospinning technology without adding any polymers [24]. The high porosity and large specific surface area of nanofibers greatly enhance their contact with the solution [25]. Hsiung [26] prepared the Ondansetron/Cyclodextrin inclusion complex nanofibrous webs (ODS/HPβCD NW) without polymers by using electrospinning technology, which improved the thermal stability and water solubility of Ondansetron. ODS/HPβCD NW could disintegrate rapidly (about 2 s) in artificial saliva, which was expected to be a rapid oral drug delivery system. The perillaldehyde/HPγCD inclusion complex was prepared into nanofibers (PA/HPγCD-IC-NFs) by using electrospinning technology [27]. PA/HPγCD-IC-NFs had high water solubility with enhanced thermal stability, antioxidant, and antibacterial performance of PA. In recent years, electrospinning technology has been gradually applied to agriculture, such as pesticide delivery, pesticide detection, and so on [21,28].

**Scheme 1:**
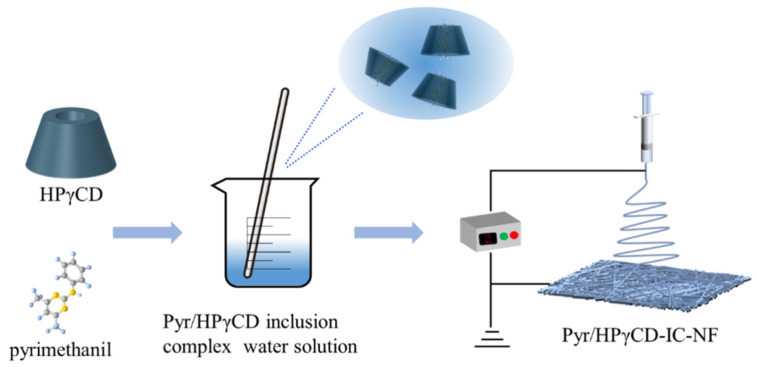
Illustration of the Pyr/HPγCD-IC-NFs synthesis process.

Pyrimethanil (Pyr) as a low toxicity fungicide of the aniline pyrimidine group is widely used for the prevention and control of gray mold in crops and ornamental plants [29], however, poor water solubility hinders its further development. Based on the above studies, it is reasonable to hypothesize that the formation of pyrimethanil/hydroxypropyl-gamma-cyclodextrin inclusion complex nanofibers (Pyr/HPγCD-IC-NFs) can effectively improve the physicochemical properties and bioactivity of Pyr. In this study, Pyr was selected as a guest molecular model of hydrophobic pesticides. The water-soluble Pyr/HPγCD-IC-NFs were produced by using electrospinning technology without polymer additions (Scheme 1). The formation and structure of Pyr/HPγCD-IC-NFs were characterized by using various crucial techniques. Additionally, the antifungal test was conducted on the Pyr and Pyr/HPγCD-IC-NFs to assess their antifungal activity.

## 2. Results and Discussion

### 2.1. Morphology Analysis

**Figure 1:**
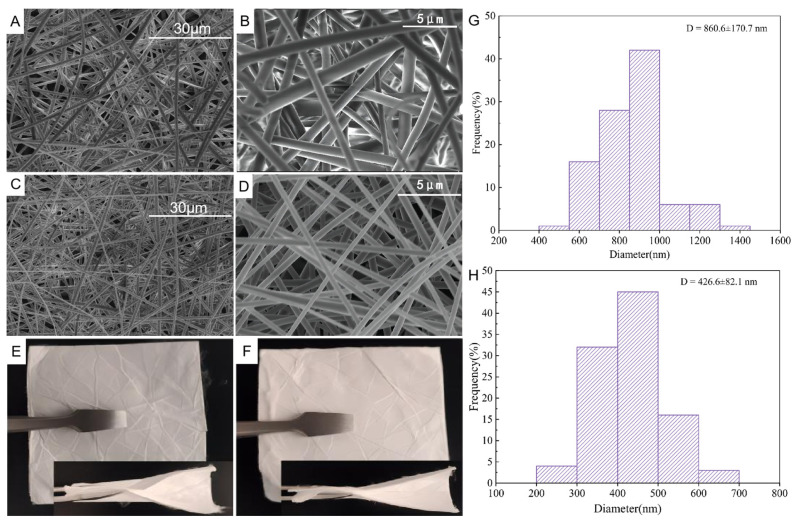
(**A**,**B**) SEM images of HPγCD NFs; (**C**,**D**) SEM images of Pyr/HPγCD-IC-NFs; (**E**) Morphology of HPγCD NFs; (**F**) Morphology of Pyr/HPγCD-IC-NFs; (**G**) Diameter distribution of HPγCD NFs; (**H**) Diameter distribution of Pyr/HPγCD-IC-NFs.

The SEM images revealed morphology of the prepared nanofibers (Figure 1). The average fiber diameter of HPγCD NFs and the Pyr/HPγCD-IC-NFs were measured to be 860.6 ± 170.7 nm and 426.6 ± 82.1 nm, HPγCD aqueous solution viscosity and conductivity were 0.69 ± 0.05 mPa•S and 6.57 ± 0.36 μs/cm, and the viscosity and conductivity of Pyr/HPγCD inclusion solution viscosity were 0.46 ± 0.06 mPa•S and 13.69 ± 0.57 μs/cm. This suggested that the viscosity and conductivity of the spinning solution affect the diameter of the electrospinning nanofibers. The optimized parameters of electrospinning result in smooth surface and good orientation nanofibers; generally, lower viscosity and higher conductivity lead to smaller fiber diameters [30]. SEM images showed that the nanofibers prepared under these electrospinning parameters have good morphology, smooth surface, and no beads. It could be clearly seen in Figure 1E,F that the prepared HPγCD NFs and Pyr/HPγCD-IC-NFs fiber membranes could be easily folded in half with certain mechanical integrity and flexibility.

### 2.2. Analysis of FT-IR Spectrum

**Figure 2:**
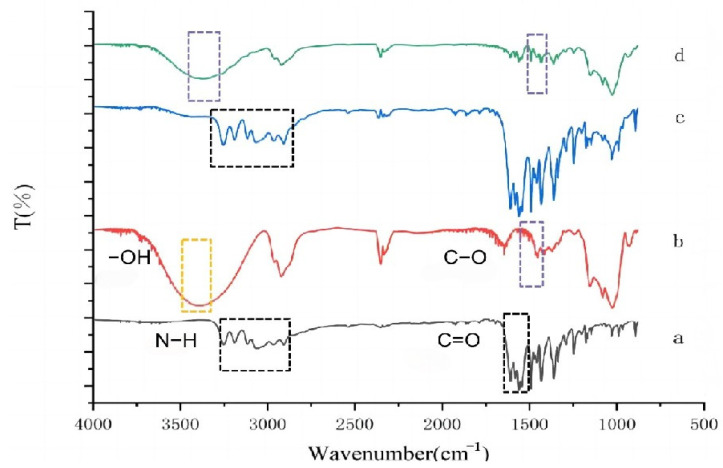
FT-IR spectra of (**a**) Pyr, (**b**) HPγCD, (**c**) Pyr-HPγCD mixtures, and (**d**) Pyr/HPγCD-IC-NFs.

The as-prepared Pyr/HPγCD-IC-NFs were successfully synthesized via the electrospinning method. FT-IR spectrum of Pyr, HPγCD-NFs, and Pyr/HPγCD-IC-NFs were presented in Figure 2. Typical absorption peaks of Pyr were mainly the stretching vibrational absorption peaks of N-H and C=N at 3200 cm^−1^ and 1624 cm^−1^. The FT-IR spectrum of HPγCD exhibits three distinct regions: a broad peak at 3412 cm^−1^ attributed to the hydrogen-bonded -OH stretching vibrations of cyclodextrin molecules; the absorption peaks at 2927 cm^−1^ corresponding to the stretching vibrations of CH_2_ and CH groups in the cyclodextrin structure; and a sharp peak near 1015 cm^−1^ originating from the stretching vibration of the C-O-C glycosidic bond, reflecting the cyclic polysaccharide backbone of HPγCD. Notably, in the spectrum of the Pyr/HPγCD inclusion complex, the characteristic N-H peak of Pyr (3200 cm^−1^) completely disappeared, and the intensity of the C=N peak significantly decreased. This phenomenon indicates that Pyr molecules are fully encapsulated within the hydrophobic cavity of HPγCD through hydrophobic interactions, with their polar groups form intermolecular hydrogen bonds with the hydroxyl groups of cyclodextrin. Consequently, their characteristic vibrational signals are masked by the strong polar absorption peaks of HPγCD [31]. Celebioglu et al. used FTIR characterization to demonstrate the formation of curcumin/cyclodextrin inclusion complexes by observing the disappearance, weakening, and shifting of some characteristic peaks of curcumin in curcumin/cyclodextrin nanofibers [32]. The FT-IR spectra of the Pyr/HPγCD inclusion complex and Pyr/HPγCD-IC-NFs were basically the same; this confirmed that the high-voltage electric field during electrospinning does not disrupt the host–guest inclusion structure, preserving the cavity integrity of HPγCD and the intermolecular interactions. Such spectral consistency further verifies the thermodynamic stability of the supramolecular inclusion complex during nanofiber formation.

### 2.3. XRD Analysis

**Figure 3:**
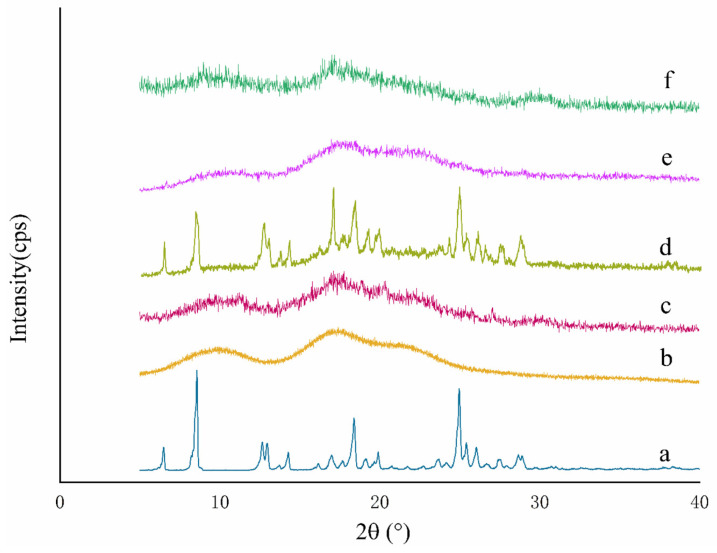
XRD patterns of (**a**) Pyr, (**b**) HPγCD, (**c**) HPγCD nanofibers, (**d**) Pyr-HPγCD physical mixtures, (**e**) Pyr/HPγCD inclusion complexes, and (**f**) Pyr/HPγCD-IC-NFs.

XRD is a powerful technique to verify the formation of Pyr/HPγCD inclusion complex nanofibers [33]. The XRD pattern (Figure 3) revealed that the key characteristic diffraction peaks of Pyr were located at 6.5°, 8.5°, 17.0°, and 28.9°, indicating that Pyr molecules existed in a highly ordered crystalline state. The XRD patterns of HPγCD nanofibers and Pyr/HPγCD-IC-NFs displayed broad and coarse arc peaks with the similar trend, which demonstrated that the crystal diffraction of samples were amorphous structures. Notably, the four characteristic Pyr diffraction peaks (6.5°, 8.5°, 17.0°, and 28.9°) completely disappeared in the XRD pattern of Pyr/HPγCD-IC-NFs, which further confirmed that Pyr molecules were fully encapsulated within the hydrophobic cavity of HPγCD [34]. This observation aligned with findings reported by Sharif et al. [35]. During the electrospinning process, host–guest interactions between HPγCD’s cyclic cavity and Pyr molecules formed inclusion complexes, where guest molecules became dispersed and isolated, preventing self-aggregation into crystalline structures [32]. The nearly identical XRD patterns of Pyr/HPγCD-IC-NFs and Pyr/HPγCD inclusion complexes demonstrated that the host–guest inclusion interaction remained stable during fiber formation, and electrospinning caused no structural damage to the complexes. In summary, XRD analysis revealed the successful formation of Pyr/HPγCD-IC-NFs.

### 2.4. TGA Analysis

**Figure 4:**
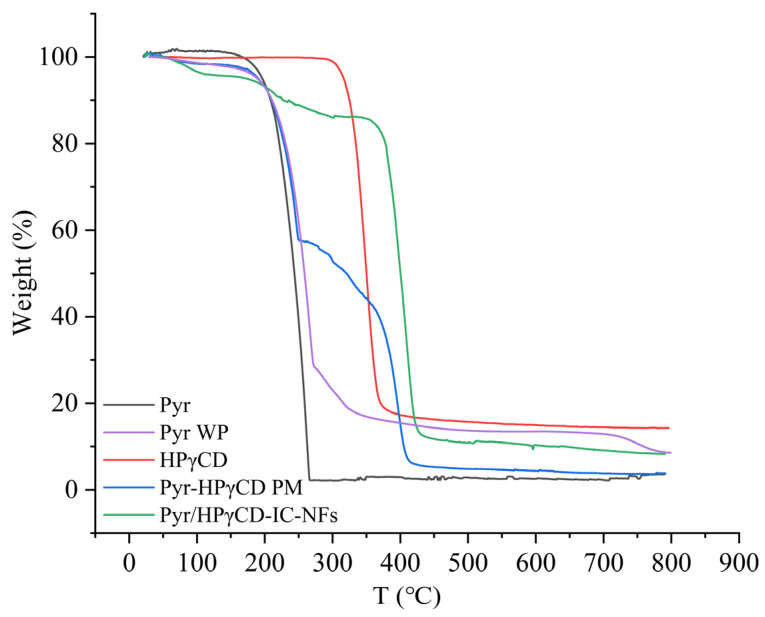
TGA curves of Pyr, HPγCD-NFs, Pyr-HPγCD physical mixtures, and Pyr/HPγCD-IC-NFs.

TGA as a method for investigating the thermal stability of samples further verified the formation of Pyr/HPγCD inclusion complexes (Figure 4). TGA curve of Pyr displayed that Pyr had only one weight loss stage at 150–266 °C, accompanied by a weight loss rate of approximately 98%. The weight loss stage of HPγCD was divided into two steps: the decrease in crystalline water at 49–90 °C and the thermal decomposition at 303–373 °C. The TGA curve of the Pyr-HPγCD physical mixture showed three weight loss stages: the evaporation of water from crystal (50–97 °C), the thermal decomposition of Pyr (170–251 °C), and the thermal decomposition of HPγCD (351–418 °C). As can also be seen from the figure, Pyr WP loses weight in the second stage at 160–270 °C, which was attributed to the thermal decomposition of Pyr, and Pyr WP did not significantly improve the thermal stability of Pyr. The Pyr/HPγCD-IC-NFs had three weight loss stages: the first weight loss occurs at 50–109 °C due to dehydration; the second weight loss (187–306 °C) was mainly due to the thermal decomposition of Pyr—it can be observed that the thermal decomposition temperature of Pyr in inclusion fiber was higher than that of Pyr and physical mixture; the third weight loss stage was at 357–430 °C, due to the thermal decomposition of HPγCD. The final weight loss ratio was 87%. Compared to pure Pyr and physical mixtures, Pyr/HPγCD-IC-NFs exhibited a wider decomposition temperature range and lower decomposition rate, which meant that the thermal stability of Pyr was enhanced by the encapsulation of HPγCD nanofiber [36]. In this way, Pyr can be prevented from decomposing in the environment prematurely when applied in the field, so as to maintain the activity of pesticides and improve the utilization rate of pesticides.

### 2.5. ^1^H NMR Spectrum Studies

**Figure 5:**
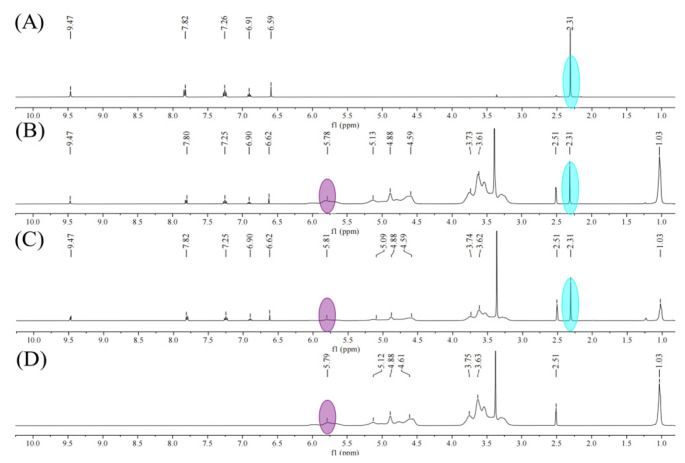
^1^H NMR spectrum of (**A**) Pyr, (**B**) Pyr/HPγCD inclusion complexes, (**C**) Pyr/HPγCD-IC-NFs, and (**D**) HPγCD.

The ^1^H NMR spectrum of the samples were displayed in Figure 5. The changes in the proton chemical shift of samples were summarized in Table 1. From the ^1^HNMR spectra of the Pyr/HPγCD inclusion complex and Pyr/HPγCD nanofibers, it could be concluded that HPγCD (5.78 ppm, 5.81 ppm) and Pyr (2.31 ppm, 2.31 ppm)—the stoichiometric ratios of which were 1:0.81 and 1:0.93, respectively—indicated that 93% of pyrimethanil was encapsulated in Pyr/HPγCD nanofibers. The OCH_2_ of HPγCD and the proton peaks of H-1, H-3, H-4, and H-5 in Pyr/HPγCD nanofibers moved to higher field, indicating that Pyr entered the cavity of HPγCD and increased the electron cloud density of protons in the cavity of HPγCD, resulting in displacement of these H of HPγCD. In Pyr/HPγCD nanofibers, H-c and H-d of Pyr moved to high field while H-e moved to low field, indicating that Pyr entered the cavity of HPγCD and H of the two interacted.

### 2.6. Phase Solubility Studies

**Figure 6:**
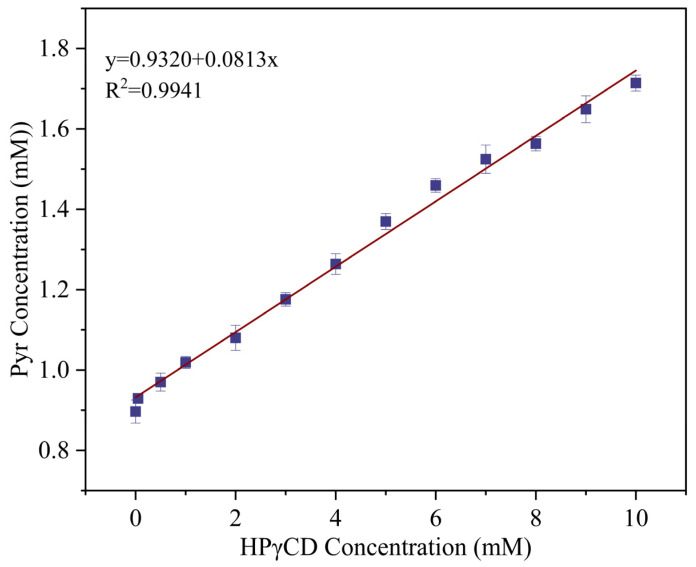
Phase solubility diagram of Pyr and HPγCD inclusion complex nanofibers.

A phase solubility curve was used as the most intuitive method to study phase solubility [37]. According to Figure 6, R^2^ of the linear fitting curve was 0.9932, and since the water solubility of Pyr and concentration of HPγCD in the solution display a positive relation, the phase solubility curves of Pyr and HPγCD conformed to AL-type. The AL-type phase solubility plot showed that guest solubility increases linearly with cyclodextrin concentration, and that Pyr formed an inclusion complex with HPγCD with a stoichiometric ratio of 1:1 (HPγCD:Pyr). For the Pyr/HPγCD inclusion complex, the Ks and CE values obtained by calculating the slope of the phase solubility diagram were 96 M^−1^ and 0.089, respectively. Complexation efficiency (CE) can be used to evaluate the ability of cyclodextrin to form inclusion complexes with hydrophobic molecules. Higher CE value indicates that cyclodextrin had a stronger inclusion ability and solubilization ability for hydrophobic guest molecules. In general, CE values of cyclodextrins and drugs in water systems basically did not exceed 1.5. The solubility of Pyr in 10 mM HPγCD solution was 1.9 times higher than that in water. In conclusion, the inclusion complex formed by HPγCD and Pyr can increase the solubility of Pyr in water significantly.

### 2.7. Determination of Dissolved Effect

**Figure 7:**
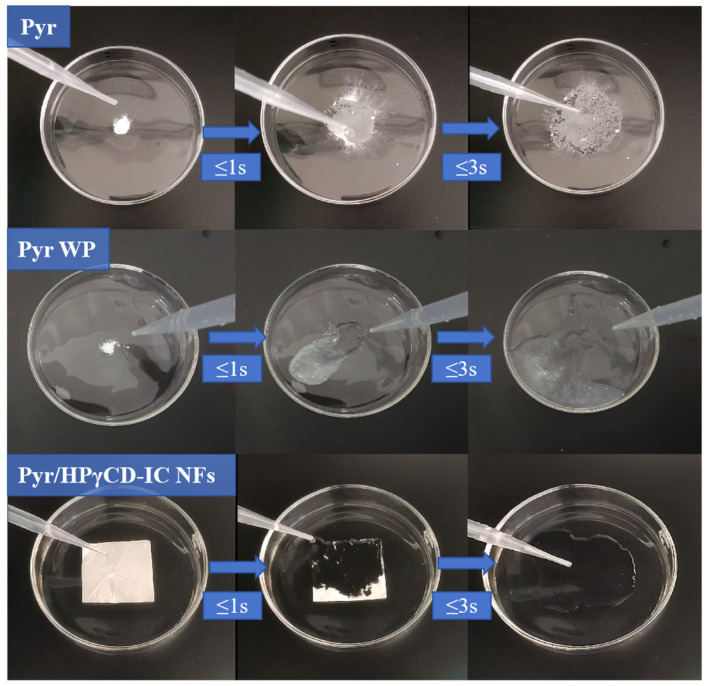
Dissolution effect images of the Pyr, Pyr WP, and Pyr/HPγCD-IC-NFs.

The water solubility of Pyr/HPγCD inclusion complex nanofibers was studied by using the rapid dissolution method [32]. As shown in Figure 7, Pyr had poor water solubility and could not be dissolved after adding water. Pyr WP also had poor water solubility, and it could not be completely dissolved within 3 s, but only partially dispersed in water. Pyr/HPγCD-IC-NFs could completely dissolve in aqueous solution within three seconds, proving that Pyr/HPγCD nanofibers improved the water solubility of Pyr. Combined with XRD analysis, Pyr existed in an amorphous form after being encapsulated by HPγCD, and its aqueous solubility was greatly enhanced. The high surface area and large pores of nanofibers were also strongly conducive to sample dissolution. In summary, the water solubility of Pyr/HPγCD-IC-NFs was superior to that of Pyr and Pyr WP. HPγCD can assist in the rapid dispersion of Pyr in water, which, in practical applications, can avoid the need for additional organic additives, thereby reducing the pollution associated with traditional pesticides.

### 2.8. Molecular Modeling

**Figure 8:**
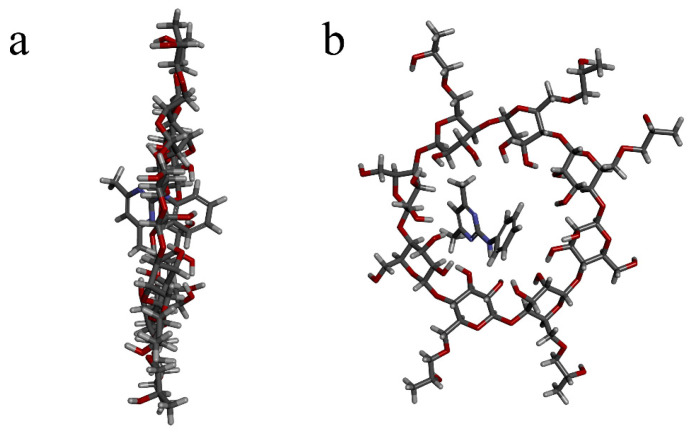
The docking modeling of Pyr and HPγCD: (**a**) lateral view, (**b**) top view.

The lateral view (a) and top view (b) of Pyr/HPγCD-IC-NFs were illustrated in Figure 8. The molecular model of Pyr and HPγCD constituting the inclusion complex was obtained by molecular modeling. The inclusion complex system as the lowest energy and the most stable structure was chosen in the simulation [38]. As shown by the docking results, the Pyr was encapsulated from the large opening of HPγCD to the HPγCD cavity, and Pyr almost entirely came into the cavity of HPγCD. According to the previous conclusions, the combination of the guest molecule Pyr and the host molecule HPγCD chiefly relied on intermolecular force. It was shown that the Pyr was encapsulated into the HPγCD cavity to form the inclusion complex.

### 2.9. Antifungal Test Analysis

**Figure 9:**
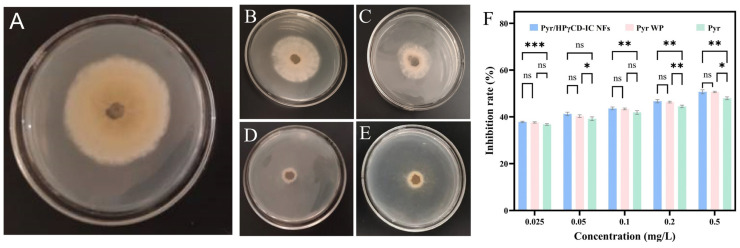
The fungicidal activities against B. cinerea of different samples: (**A**) blank control, (**B**) HPγCD NFs, (**C**) Pyr, (**D**) Pyr/HPγCD-IC-NFs, and (**E**) Pyr WP. (CPyr = 0.10 μg/mL); (**F**) inhibition rate histogram (* *p* < 0.05, ** *p* < 0.01, *** *p* < 0.001, ns *p* > 0.05).

Botrytis cinerea was employed to elucidate the antifungal activity of Pyr/HPγCD-IC-NFs through the comparison of the fungus growth diameters (Figure 9). The growth diameter of *B. cinerea* in the medium added with HPγCD NFs was slightly smaller than that in the blank group, which indicated that HPγCD had no antifungal activity. Compared with Pyr, the average growth diameter of *B. cinerea* was smaller in the medium added with Pyr WP and Pyr/HPγCD-IC-NFs, which illustrates that Pyr WP and Pyr/HPγCD-IC-NFs displayed better antifungal activity. From the histogram, it can also be observed that there is no significant difference in the inhibition rate against Botrytis cinerea between Pyr/HPγCD-IC-NFs and Pyr WP, while both show statistically significant differences compared to Pyr alone. With the forming of the inclusion complex, the water solubility of Pyr was increased, allowing the active ingredients to further exert antifungal effects. As a mature commercial formula, Pyr WP was added with cosolvent to improve the water solubility of Pyr, and its bactericidal effect was better than that of pure Pyr. Table 2 displayed the Toxicity Equations, the correlation coefficient (R), and EC_50_ of Pyr and Pyr/HPγCD-IC-NFs. The data showed that the EC_50_ value of Pyr was 1.92 times that of Pyr/HPγCD-IC-NFs, that is, the antifungal activity of Pyr/HPγCD-IC-NF was 1.92 times that of Pyr, which also proved that Pyr/HPγCD-IC-NFs could improve the fungicidal effect of Pyr. The EC_50_ value of Pyr WP was 0.459 μg/mL, which was slightly higher than that of Pyr/HPγCD-IC-NFs, indicating that the bactericidal ability of Pyr/HPγCD-IC-NFs was better than that of commercial Pyr WP. The correlation coefficient (R) and EC_50_ values of Pyr and Pyr/HPγCD-IC-NFs indicate that the prepared Pyr/HPγCD inclusion complex nanofibers can significantly enhance the utilization efficiency of Pyr. This improvement facilitates the reduction of application rates, enhances efficacy, and mitigates environmental pollution and toxic side effects on non-target organisms caused by excessive application.

## 3. Materials and Methods

### 3.1. Materials

Pyr was obtained from HaoHong Bio-Technology Company (purity 99%, Shanghai, China). HPγCD was sourced from Yuanye Bio-Technology Company (Shanghai, China). Potassium bromide (KBr, G.R., Tianjin China) was obtained from Tianjin Guangfu Technology Development Company (Tianjing, China). Pyr Wettable Powder (Pyr WP) was purchased from Rongbang Chemical Co., Ltd. (70%, Weifang, China). Potato agar was provided by Aobo Biotechnology Company (Beijing, China). All the other chemical reagents were obtained from Aladdin Company (Shanghai, China).

### 3.2. Preparation of Solutions for Electrospinning

One gram of HPγCD was dissolved in 0.5 mL of distilled water and mixed at room temperature to obtain 200% (*w*/*v*) cyclodextrin solution. The Pyr/HPγCD inclusion complex solution was produced by adding Pyr to HPγCD aqueous solution with a molar ratio of 1:1 (nPyr:nHPγCD), and the mixture was stirred for 12 h at room temperature [39].

### 3.3. Preparation of Electrospinning Nanofibers

Spinning was carried out in an electrostatic spinning machine (DT-1005, Dalian Dingtong Technology Co., Ltd., Dalian, China). First, HPγCD solution or Pyr/HPγCD inclusion solution was loaded into a 1 mL metal needle plastic syringe (the inner diameter of the metal needle was 0.4 mm). Second, the plastic syringe was mounted horizontally on the metering pump (TYD01, Baoding Leifu Fluid Technology Co.,Ltd., Baoding, China) and then pushed at 0.5 mL per hour. Third, the receiving distance was set to 10–15 cm, and the voltage was fixed to 15 kV. Finally, the nanofiber films were placed on the receiving device. They were denoted as HPγCD NFs and Pyr/HPγCD-IC-NFs respectively. It is worth noting that the experiment was in an airtight Perspex box with 18% relative humidity, and the resulting nanofibers were stored in a refrigerator [40].

### 3.4. Preparation of Pyr/HPγCD Inclusion Complex Solid

According to the method in Section 3.2, the Pyr/HPγCD inclusion complex solution was prepared. The solution was refrigerated at 1–3 °C for 12 h after stirring. The frozen solution was filtered to obtain the inclusion complex and then dried in a vacuum at 60 °C for 4 h to make the Pyr/HPγCD inclusion complex solid.

### 3.5. Preparation of Pyrimethanil-HPγCD Physical Mixture

After grinding and evenly mixing 0.001 mol of HPγCD and 0.001 mol of pyrimethanil, the combination was then dried in a vacuum at 60 °C for 4 h to obtain the pyrimethanil-HPγCD physical mixture (Pyr- HPγCD PM).

### 3.6. Measurements and Characterization

#### 3.6.1. Morphology Analysis

HPγCD NFs and Pyr/HPγCD-IC-NFs were observed by SEM (SU-8010, Hitachi, Tokyo, Japan). The samples sputter thin gold layers under a high vacuum before testing. The SEM images were taken at an accelerating voltage of 12.5 kV. The average nanofiber diameter was measured by randomly choosing 100 fibers from SEM images by the Nano measurer for calculation. The physicochemical properties of the spinning solution (e.g., conductivity, viscosity) affects the morphology of electrostatically spun nanofibers [41]. Conductivity and viscosity of the HPγCD solution and the Pyr/HPγCD inclusion complex nanofiber solution were tested at room temperature.

#### 3.6.2. FT-IR Analysis

The sample was subjected to infrared spectroscopic analysis using the potassium bromide (KBr) pellet method. The Pyr, HPγCD, Pyr/HPγCD physical mixture and the Pyr/HPγCD inclusion complex nanofibers were studied by using an IR tracer-100 spectrometer (Shimadzu, Kyoto, Japan). The resolution of the FTIR was chosen to be 4 cm^−1^, the wave number range was selected to be from 4000 to 400 cm^−1^, and the scanning frequency was set to 64.

#### 3.6.3. XRD Study

The Pyr, HPγCD, HPγCD nanofibers, Pyr/HPγCD physical mixture, Pyr/HPγCD inclusion complex, and Pyr/HPγCD-IC-NFs were characterized by an X-ray diffractometer. The X-ray source was Cu-κα (λ = 1.5406 A), and the scanning speed was set to 2°/min. The scanning start angle was set to 5° and the stop angle was set to 40° [42].

#### 3.6.4. Analysis of Thermal Stability

The thermal stability of Pyr, Pyr WP, HPγCD, Pyr/HPγCD physical mixture, and Pyr/HPγCD-IC-NFs were evaluated using a thermogravimetric analyzer under constant dry nitrogen flow, with a 10 °C min^−1^ heating rate, and a scanning temperature range from 50 °C to 800 °C [43].

#### 3.6.5. ^1^H NMR Analysis

The Pyr, HPγCD, Pyr/HPγCD physical mixture, and Pyr/HPγCD-IC-NFs were analyzed by Brooke AVANCE 300 MHZ. Firstly, instrument parameters needed to be specified, which included Deuterated dimethylsulfoxide (DMSO-d6) as a solvent. The stoichiometric ratio of Pyr versus HPγCD in the Pyr/HPγCD inclusion complex was obtained by calculating the ratio of the integral area of their proton peaks [35].

#### 3.6.6. Phase Solubility Study

Twenty-five milliliters of HPγCD aqueous solutions with a certain gradient were added to an excess amount of Pyr and vigorously agitated at 30 °C for 3 days, followed by filtration. Finally, the Pyr solubility data was recorded [44]. The inclusion equilibrium constant *Ks* was calculated according to Formula (1), which represented the binding force between Pyr and HPγCD. The k is the slope of the phase solubility graph, and b is the intercept of the phase solubility diagram.

(1) $$Ks=\frac{k}{b\left(1-k\right)}$$

The complexing efficiency (*CE*) was calculated based on Formula (2):

(2) $$CE=b\times Ks=\frac{[Pyr/HP\gamma CD]}{HP\gamma CD}=\frac{k}{1-k}$$

where [Pyr/HPγCD] represents the consistency of Pyr/HPγCD, and [HPγCD] represents the consistency of the HPγCD solution.

This experiment is repeated three times, and the results are displayed as average values and standard deviations.

#### 3.6.7. Rapid Dissolution Experiment

A certain mass of Pyr, Pyr WP, and Pyr/HPγCD-IC NFs (with the same mass of Pyr contained) was added to petri dishes. Then, 5 mL of distilled water was quickly added to each dish, and the dissolution of the samples within 3 s was observed and photographed for documentation. This experiment was repeated three times.

#### 3.6.8. Molecular Simulation

3D molecular structures of HPγCD and Pyr were built by sketchmodule of sybyl-X 2.0 package. In Discovery Studio 2.5, the choice was the “CDOCKER” docking simulation method, using the force field to eliminate water molecules before docking. During the docking process, the active sites on the cavity of cyclodextrin were precisely defined, with a subregion-ligand center distance set at 13.0 Å. The maximum hit parameter was configured at 100, while all other parameters retained their default values [45].

#### 3.6.9. Antifungal Activity

The antifungal activity of Pyr, Pyr WP, HPγCD nanofibers, and Pyr/HPγCD-IC-NFs against *B. cinerea* was studied by the mycelial growth rate method. The specific concentration gradient of fungicide solution was prepared with sterile water. The whole experiment process was conducted on a clean bench. Ten milliliters of potato glucose agar medium solution was slowly poured into the petri dish, and one milliliter of sample solution was fully mixed with the medium solution. All samples were sterilized before introducing the medium. After solidification of the medium, a mycelium ring measuring 6 mm in diameter was carefully positioned at the center, and subsequently cultured for two days at 27 °C. Culture medium without fungicide solution was applied as a control. The experiment was repeated three times for each group of samples. The growth diameter of mycelia was measured 48 h later. The growth inhibition rate was determined based on Equation (3).

(3) $$Inhibition\;rate\left(\%\right)=\frac{C-T}{C}\times 100\%$$

where C represents the average mycelium diameter (mm) of the control group, and T signifies the average mycelium (mm) diameter of the experimental group. Based on the correlation between the consistency of Pyr and the growth inhibition rate of sample, the EC_50_ values of Pyr and Pyr/HPγCD-IC-NFs were calculated.

This experiment was repeated three times, and the results were displayed as average values and standard deviations.

#### 3.6.10. Statistical Analysis

The experimental results were repeated three times, and the data processing results were represented by mean ± SD. According to the situation, one-way or two-way ANOVA was selected for statistical analysis (*p* < 0.05).

## 4. Conclusions

In this work, pyrimethanil/cyclodextrin inclusion complex nanofibers were prepared by electrospinning techniques in the absence of polymerization. The successful preparation of Pyr/HPγCD-IC-NFs was verified by FT-IR, ^1^H NMR, and XRD. Compared with Pyr, the smooth and bead-free Pyr/HPγCD-IC-NFs had better solubility, thermal stability, and fungicidal activity. The initial thermal decomposition temperature of Pyr increased from 150 °C to 187 °C. The rapid dissolution experiments demonstrated that the water solubility of pyrimethanil could be obviously improved after preparation of the inclusion nanofibers, thanks to the encapsulation of hydrophilic HPγCD and the large specific surface area of the nanofibers. The prepared inclusion nanofiber can be completely dissolved in water within 3 s. According to the antifungal test, the EC_50_ value of Pyr/HPγCD-IC-NFs (0.437 μg/mL) to *B.cinerea* was slightly lower than that of Pyr WP (0.459 μg/mL), and their bactericidal ability was stronger than that of pure Pyr (0.437 μg/mL). The Pyr/HPγCD-IC-NFs significantly enhances the water solubility of Pyr without the addition of organic adjuvants, providing a viable solution to mitigate pollution caused by organic additives in traditional commercial formulations. This approach also offers an eco-friendly and feasible method to improve the usability of hydrophobic pesticides. Gray mold exhibits a high incidence rate in fruits, occurring throughout the entire process of fruit growth, harvesting, storage, and transportation. The findings of this study can be applied either as a direct spray solution, a medicated lichen formulation, or fruit bagging materials to prevent and control gray mold disease. In the future, it is necessary to carry out field experiments on Pyr/HPγCD-IC-NFs to broaden the application prospect of Pyr/HPγCD-IC-NFs.

## Figures and Tables

**Table 1:** Chemical shifts of inclusion complexes and their nanofibers related to Pyr and HPγCD.

	Chemical Shift (δ)							
	OCH2	H-1	HOC H_2_	H-3	H-5	H-4	H-2	CH_3_
HPγCD	5.79	5.12	4.88	4.61	3.75	3.63	2.51	1.03
Pyr/HPγCD NFs	5.81	5.09	4.88	4.59	3.74	3.62	2.51	1.03
∆δ	−0.03	0.03	0.00	−0.02	−0.01	−0.01	0.00	0.00
	**Chemical Shift (δ)**							
	**H-a**	**H-b**	**H-c**	**H-d**	**H-e**	**H-f**		
Pyr	9.47	7.82	7.26	6.91	6.59	2.31		
Pyr/HPγCD NFs	9.47	7.82	7.25	6.90	6.62	2.31		
Δδ	0.00	0.00	−0.01	−0.01	0.03	0.00		

**Table 2:** The toxicological equations of Pyr and Pyr/HPγCD inclusion complex nanofibers against B. cinerea and EC_50_.

Samples	Toxicological Equation	Correlation Coefficient (R)	EC_50_ (μg/mL)	Increased Multiple
Pyr	Y = 0.2207x + 5.0161	0.9999	0.840	-
Pyr WP	Y = 0. 2507x + 5.0848	0.9997	0.459	1.84
Pyr/HPγCD-IC- NFs	Y = 0.2485x + 5.0893	0.9999	0.437	1.92

## Data Availability

The original contributions presented in this study are included in the article. Further inquiries can be directed to the corresponding author(s).
